# OM-85 BV in pediatric recurrent respiratory tract infections: a cost-utility analysis

**DOI:** 10.1186/s12890-022-02264-9

**Published:** 2022-12-06

**Authors:** Jefferson Antonio Buendía, Diana Guerrero Patiño, Erika Fernanda Lindarte

**Affiliations:** 1 Research Group in Pharmacology and Toxicology “INFARTO”, Department of Pharmacology and Toxicology, University of Antioquia, Medellín, Colombia; 2 Facultad de Medicina, Universidad de Antioquia, Carrera 51D #62-29, Medellín, Colombia

**Keywords:** Health economics, Public health, Healthcare, Colombia, Corticosteroids

## Abstract

**Background:**

Despite the growing evidence on efficacy, little is known regarding the cost-utility of Vaxom/Imocur (OM-85 BV) supplementation to decrease the probability of recurrent respiratory tract infections in OM-85 BV to reduce the incidence of recurrent respiratory tract infections in children.

**Methods:**

A decision tree model was used to estimate the cost and quality-adjusted life-years (QALYs) of OM-85 BV in a patient aged 1–6 with a history of recurrent respiratory tract infections. Multiple sensitivity analyses were conducted to evaluate the robustness of the model. Cost-effectiveness was evaluated using the willingness-to-pay defined for Colombia of US$5180 per QALY. The time horizon defined was six months. Costs were estimated from a societal perspective.

**Results:**

The expected annual cost per patient with OM-85 BV was US$843 and with placebo was US$1167. The QALYs per person estimated with OM-85 BV was 0.91 and with placebo was 0.89.

**Conclusion:**

In conclusion, our study shows that OM-85 BV is a cost-effective strategy to reduce the incidence of recurrent respiratory tract infections in children. Our study provides evidence that should be used by decision-makers to improve clinical practice guidelines.

## Background

Recurrent respiratory tract infections (RTI) are frequent events that generate a high burden of morbidity in childhood. These RTI (acute otitis media occurs > 4 times per year, sore throat > 5 times per year, or otitis media with effusion continues for > 6 months or more than one episode of community acquired pneumonia pneumonia in a single year) can affect about 25% of infants under 1 year old and 6% in the first 6 years of life [1]. Despite being a condition that is likely to improve with age, RTI can cause significant medical, social, and economic problems for the child and society [1, 2]. RTI was associated with lower health-related quality of life in both children and their caregivers [3]. Children with RTI showed substantively lower physical, emotional, social, and school functioning scores than healthy children and their caregivers also scored lower on physical, emotional, social, cognitive, and communication functioning [3]. In addition to the preventive measures already proven to reduce the incidence of RTI, such as reducing exposure to second-hand smoke, reducing exposure to indoor and outdoor pollutants, improving hand washing, promoting breastfeeding, adequately vaccinating of children, bacterial immunomodulators have been proposed as a preventive intervention [4].

Broncho-Vaxom/Imocur (OM-85 BV) is an immunostimulant used to prevent RTI containing multiple Toll-like receptor-like ligands [4]. This immunostimulant contains 3.5 mg per capsule of a lyophilized fraction of alkaline lysis of 21 strains of 8 species of common respiratory tract pathogens: *Haemophilus influenza, Streptococcus pneumoniae, Klebsiella pneumoniae, Klebsiella ozaenae, Staphylococcus aureus, Streptococcus pyogenes, Streptococcus viridans and Neisseria catarrhalis*. In a recent systematic review and meta-analysis of 14 studies in 1151 children with RRI, OM-85 BV was associated with lower total duration of respiratory tract infections (mean difference (MD) − 19.51; 95% CI − 23.00 to − 16.01, *P* < 0.001); lower incidence of respiratory tract infections (OR 0.40; 95% CI 0.21–0.77, *P* = 0.006); and lower antibiotic use (OR 0.38; 95% CI 0.29–0.52, *P* < 0.001) with no significant difference in adverse event rate (OR 1.02; 95% CI 0.52–2.03, *P* = 0.94) [5].

The Inter-society Consensus of Prevention of recurrent respiratory infections in 2021, regarding this drug states: “Among the lysates, OM-85 BV has demonstrated a consistent likelihood of efficacy and can be recommended in selected populations of children, always considering the cost–benefit ratio” [4]. However, to date, no economic evaluations have been published in developing countries. The contribution of an economic evaluation to the current evidence lies not only in estimating whether it is cost-effective, but also in determining other results that are inputs for estimating the impact of such an intervention on public health, such as the cost-savings per patient treated with this drug. The objective of the present study was to estimate the cost-utility of OM-85 BV to reduce the incidence of recurrent respiratory tract infections in children.

## Methods

### Base case

**Fig. 1:**
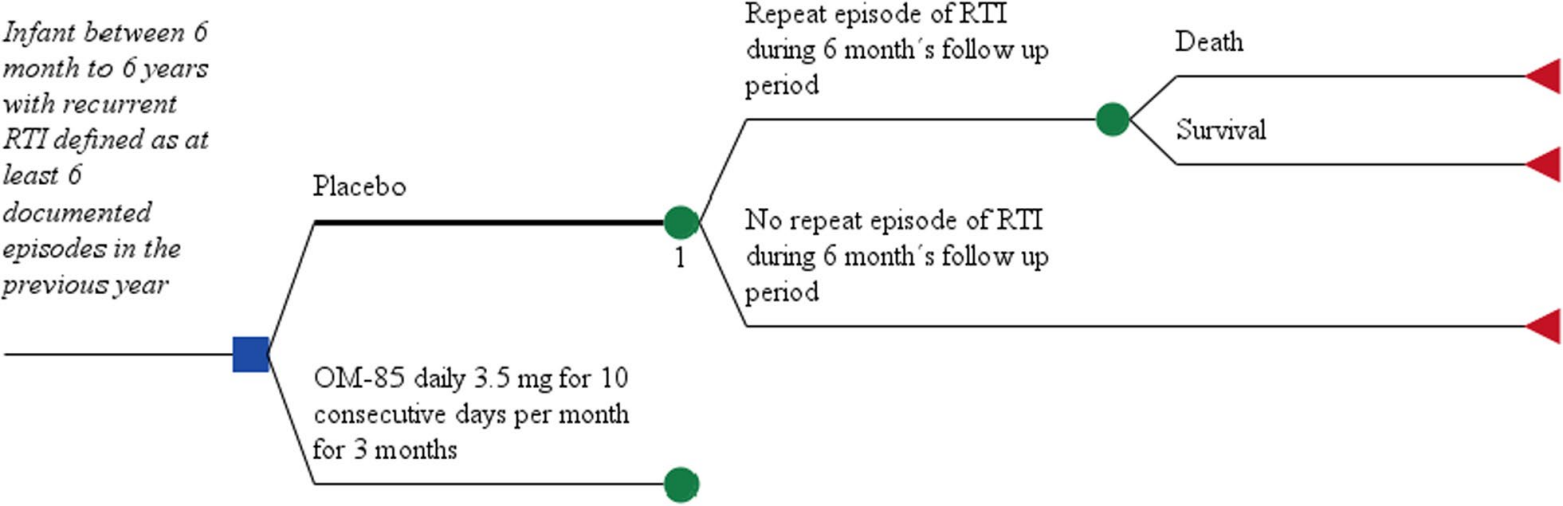
Decision tree model

A decision tree model was used to estimate the cost and quality-adjusted life-years (QALYs) of OM-85 BV as a preventive treatment of RTI. It was decided to use a decision tree model because we are going to model interventions (OM-85 BV vs placebo) with an outcome (the incidence of new episodes of RTI at six months after of the onset of this drug) that can be measured at a specific time point. This decision tree model was constructed according to the natural history of RTI, Fig. 1. The base case corresponds a patient aged 1–6 years with a history of recurrent RTIs, defined as at least 6 documented episodes in the previous year. The decision tree begins with a decision node and two options: 3.5 mg of OM-85 BV per once a day for the first 10 days of each the first 3 months or placebo with the same scheme of administration for the second 3 months. Then, in each branch, there are two possibilities that the patient dies or survives this new episode of RTI. The only difference between the two decision branches is the probability of a new episode of RTI at 6 months.

The time horizon defined was six months. Given the short time horizon, no discount rates were applied to costs or QALYs. Cost-effectiveness was evaluated at a willingness-to-pay (WTP) value of US$5180 [6]. Data on mortality from CAP and recurrent pneumonia were obtained from local data reported by national surveillance of acute respiratory infections and national vital statistics [7–9]. The relative risk and probability of RTI at 6 months were extracted from systematic review and meta-analysis of 14 randomized controlled trials with 1859 patients that found that OM-85 BV, relative to the placebo group, was significantly related to lower incidence of respiratory tract infections (OR 0.40; 95% CI 0.21–0.77, *P* = 0.006) without differences in adverse event rate (OR 1.02; 95% CI 0.52–2.03, *P* = 0.94); [5].

Utilities were extracted from a utility assessment study of parent preferences for pediatric health outcomes [10], Table 1. This study was conducted in 4016 parents or guardians at least 18 years of age with at least 1 child under age 18 years, each subject's utilities were assessed on 3 random health states out of 29 chosen for the study. We assumed that the utility of the new RTI state is equal to the utility reported for the 10-day hospitalization state in this study, given that the worst-case scenario of a new RTI, such as uncomplicated pneumonia, requires hospitalization rather than outpatient management. For the patient who did not develop RTI, it was assumed that the utility of this condition is equal to that reported for uncomplicated acute otitis media; given that the base case are patients with previous RTI and were not in full health during the previous 6 months. Both the time trade-off and standard gamble methods were used to measure utilities.

**Table 1:** Model inputs

Model input	Base case value	Distribution
*Probabilities*		
Repeat episode of RTI	0.20	β (SD: 0.05)
Mortality repeat episode of RTI	0.008	β (SD: 0.001)
*Utility*		
Case base	0.94	β (SD: 0.01)
Repeat episode of RTI	0.87	β (SD: 0.2)
*Cost*		
Repeat episode of RTI (US$)	2022	Γ (SD: 505)
OM-85 BV × 3 Cycles of 10 days (US$)	74	Γ (SD: 18,5)
*OM-85 BV*		
Reduction of probability of repeat RTI	0.65	LogN (SD: 0.07)

*RTI* respiratory tract infections

Since utilities and relative risks do not come from the Colombian population, they were subjected to probabilistic sensitivity analysis as detailed below as recommended by the Consolidated Health Economic Evaluation Reporting Standards (CHEERS) Statement [11]. The costs were estimated from a societal perspective (including direct and indirect costs). All direct and indirect costs were extracted from a previously published cost-of-illness study in children with pneumonia in Colombia [12]. This study estimates the direct medical costs and indirect non-medical costs of pneumonia in children under 5 years, based on information obtained from 275 patients hospitalized using the databases of the Individual Registry of Services Provision and Sufficiency and a survey constructed, validated, and applied to parents, mothers, and caregivers of children hospitalized by these events [12]. Drug costs were taken from the National Drug Price Information System [13]. All cost costs were transformed to 2021 costs using official inflation data in Colombia. We used US dollars (Currency rate: US$ 1.00 = COP$ 3,900) to express all costs in the study [8]. The incremental cost-effectiveness ratio (ICER) was calculated using the following equation:

$$ICER = \frac{{\begin{array}{*{20}c} {{\text{Expected}}\;{\text{annual}}\;{\text{cost}}\;{\text{per}}\;{\text{patient}}\;{\text{with}}\;{\text{OM}} - 85\;BV - } \\ {{\text{Expected}}\;{\text{annual}}\;{\text{cost}}\;{\text{per}}\;{\text{patient}}\;{\text{without}}\;{\text{OM}} - 85\;BV} \\ \end{array} }}{{\begin{array}{*{20}c} {QALY\;per\;patient\;with\;OM - 85\;BV - } \\ {QALY\;per\;patient\;without\;OM - 85\;BV } \\ \end{array} }}$$

Also, we estimated the net monetary benefit (NMB). NMB represents the value of an intervention in monetary terms [14]. NMB is calculated as (incremental benefit x threshold)—incremental cost. Incremental NMB measures the difference in NMB between alternative interventions, a positive incremental NMB indicating that the intervention is cost-effective compared with the alternative at the given willingness-to-pay threshold.

### Sensitivity analysis

We conduct a one-way sensitivity analysis presenting these results in a tornado diagram. The ranges of utilities, transition probabilities and costs were estimated assuming a variability of plus or minus 25%. The relative risk range corresponded to the published confidence interval of the value taken from the literature described above. Probabilistic sensitivity analysis was also performed. For this purpose, random sampling was performed from each of the parameter distributions. We used the log normal for relative risk, beta distribution for utilities and the gamma distribution for costs, see Table 1. For each treatment strategy, we calculated the expected costs and QALYs using the combination of all parameter values in the model. To do this calculation, a second-order Monte Carlo simulation with 10,000 replications of each parameter was made: resulting in the expected cost-utility for each treatment strategy. To represent decision uncertainty, we plot the frontier of the cost-effectiveness acceptability curve [15]. TreeAge Pro Healthcare 2022 software^®^ was used in all analyses.

## Results

The main results are presented in Table 2. The base-case analysis showed that compared with placebo, OM-85 BV was associated with lower costs and higher QALYs. The expected annual cost per patient with OM-85 was US$843 and with placebo was US$1167. The QALYs per person estimated with OM-85 was 0.91 and with placebo was 0.89. The NMB with OM-85 BV was US$ 4119 and with placebo was US$ 3724. This position of absolute dominance (OM-85 has lower costs and higher QALYs than placebo) of OM-85 BV it is unnecessary to estimate the incremental cost-effectiveness ratio.

**Table 2:** Cost effectiveness analysis

Strategy	Cost (US$)	Diff ($)	QUALYs	Diff (QALYs)	NMB($)
OM-85 BV	843		0.91		4119
PLACEBO	1167	594	0.89	0.01	3724

### Sensitivity analysis

**Fig. 2:**
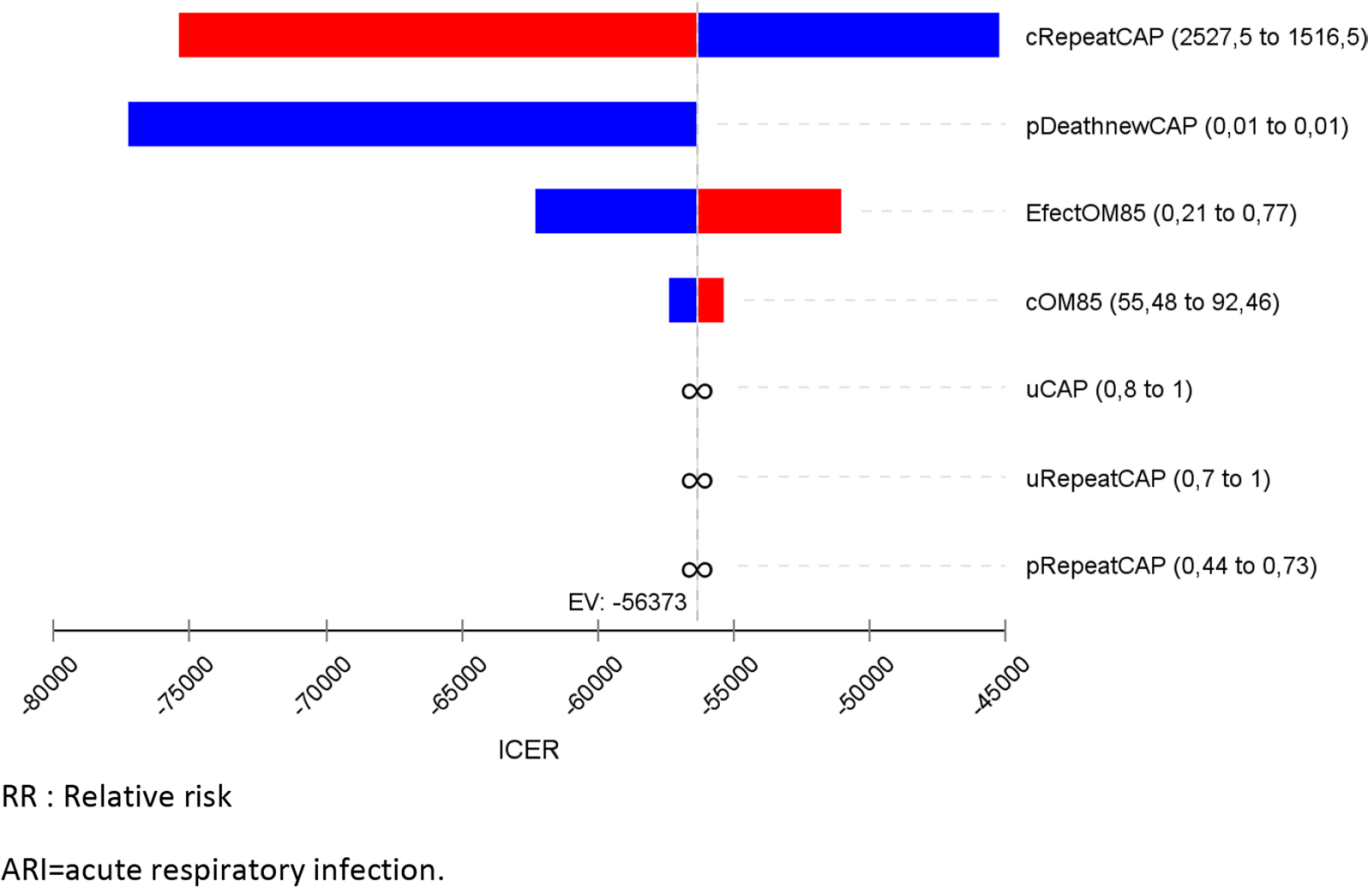
Tornado diagram. RR: Relative risk, ARI = acute respiratory infection

**Fig. 3:**
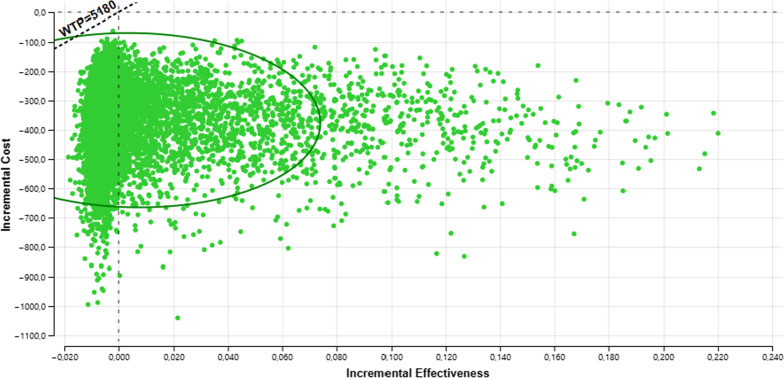
Cost effectiveness plane

**Fig. 4:**
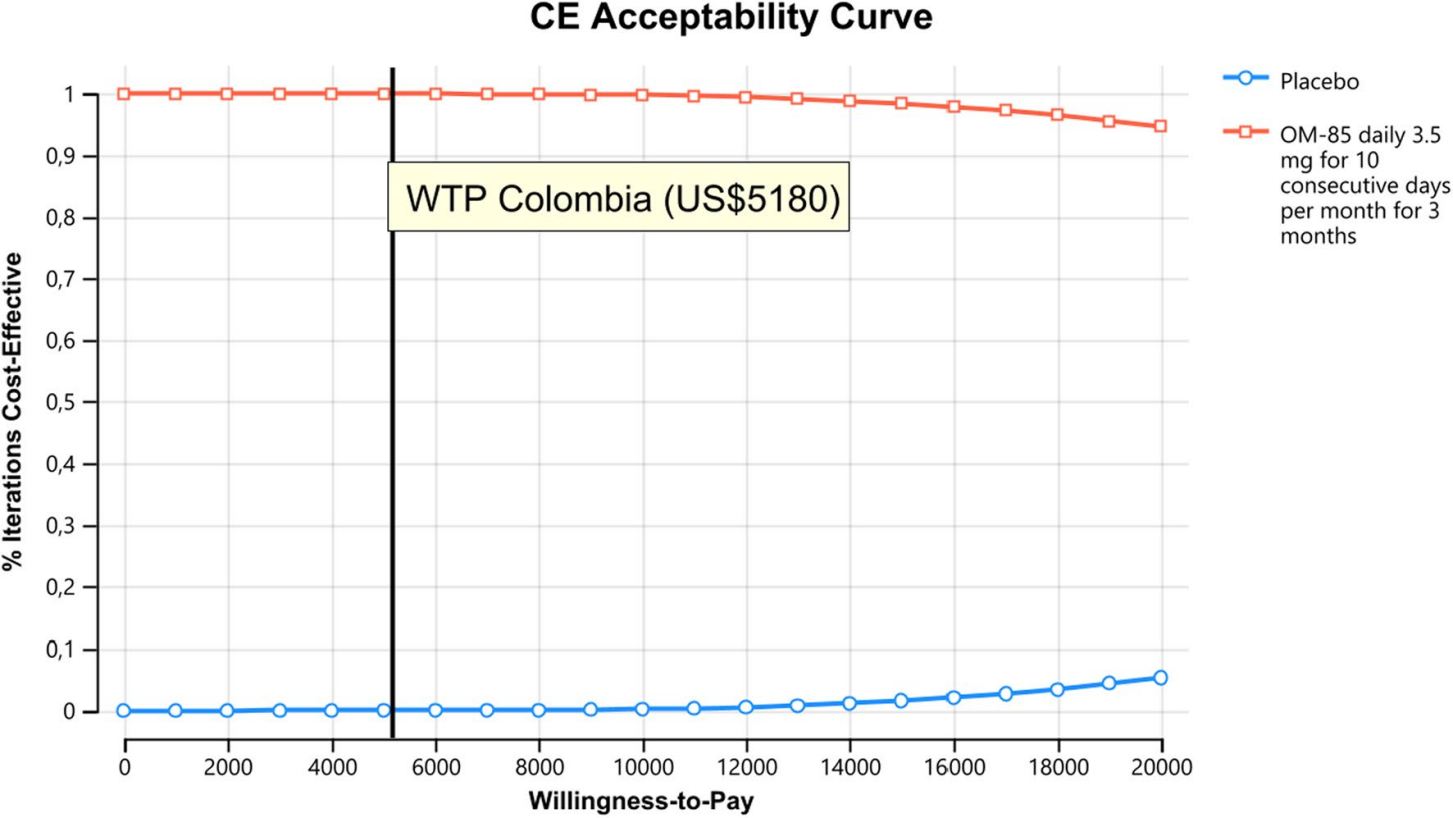
Acceptability curve

In the deterministic sensitivity analyses, our base‐case results were robust to variations in utilities, transition probabilities, and cost; Fig. 2. That is, changing each of the parameters, within the ranges mentioned in the methods section, of cost, utilities, probabilities and relative risk did not alter the incremental cost-effectiveness ratio substantively or change its interpretation. The results of the probabilistic sensitivity analysis are graphically represented in the cost-effectiveness plane, Fig. 3. This scatter plot shows that 99% of simulations the ICER were below WTP in quadrants 2 (42%) or 3 (57%). The incremental net monetary benefit (INMB) calculated in the second-order Monte Carlo simulation was US$395. This positive value of INMB means that the incremental benefits in monetary terms for the WTP are higher than incremental costs of this drug in Colombia; thus, this medication can be declared as cost-effective. For WTP in Colombia (US$5180 per QALY), there is a 100% probability that OM-85 is cost-effective at the WTP threshold. As can be seen in the acceptability curve Fig. 4.

## Discussion

Our economic evaluation shows that OM-85 BV is cost-effective to reduce the incidence f recurrent respiratory tract infections in children in Colombia. Evaluating treatments to reduce costs and optimize health resources is a priority for all health systems, especially in RTI, which, due to their frequency, generate a high economic burden in developing countries. In our study, OM-85 BV was a strategy that generated savings US$324 (UI 95% US$315–US$324) per patient, which is not insignificant given the frequency of RTI in most developing and developed countries.

Our results are in line with previous economic evaluations of OM-85. Xuan et al. [16], using cost-effectiveness decision tree model compare OM-85 BV with placebo therapy for managing the acute exacerbation of chronic bronchitis and rhino sinusitis in the Chinese population from a Chinese payer perspective. OM-85 BV is a cost-effective therapy in China. OM-85 BV, when compared with placebo arm, can prevent one additional episode exacerbation of rhino sinusitis with only RMB 1182.84 extra costs, below of WTP in China. Troiano et al. [17], in a systematic review and metanalysis of 13 studies found that OM-85 BV is associated with a reduction in the mean number of COPD exacerbations (*P* < 0.01; WMD =  − 0.86; CI 95% − 1.38, − 0.34) and in the days of antibiotic therapy (*P* < 0.01; WMD =  − 9.49; CI 95% − 11.93, − 7.05). Pessey et al. [18] using a decision-analysis model, evaluated the pharmacoeconomic impact for the French Social Security System of preventing recurrent acute rhino pharyngitis in at-risk children with OM-85 BV. Using OM-85 BV prevention, 1.52 infections were prevented in 6 months saving 67.83 Euro on the costs of care for the recurrently infected child. OM-85 BV was cost-effective in preventing acute respiratory infection (ARI) and also showed cost savings in over 70% of cases for direct costs with a reduction of 2.61 episodes of ARI during a follow-up of six months [19]. These results with OM-85 BV have been similar to those obtained in other countries such as France and Italy [18, 20, 21].

There is no consensus concerning interventions to prevent RTI. Reducing exposure to damp and mold, for example, is the intervention for which a good-quality systematic review supports the elimination of this risk factor for recurrent respiratory acute infections [4]. Other environmental interventions with low or very low-quality studies are also recommended, such as discouraging exposure to second and third-hand smoke and pollutants in general, in addition, to improving hand washing as one of the best methods to reduce respiratory infections [4, 22]. Our study provides further evidence regarding the efficiency of use of this OM-85 BV in children with RTI, which complements the growing evidence of effectiveness and safety of this drug.

Our study has some limitations. We use relative risk, utilities; transition probabilities were extracted from the literature and not estimated directly from our population. As was mentioned previously, the reliability and robustness of the results were evaluated by sensitivity analysis. The changing each of these parameters, within their ranges did not alter the incremental cost-effectiveness ratio substantially or change its interpretation. The results of this economic evaluation given the base case are only applicable to children. The direct medical cost was obtained from a retrospective study published previously from Colombia and cannot exclude selection or information bias in these values.

## Conclusion

In conclusion, our study shows that OM-85 BV is a cost-effective strategy to reduce the incidence of recurrent respiratory tract infections in children. Our study provides evidence that should be used by decision-makers to improve clinical practice guidelines.

## Data Availability

The datasets generated and/or analyzed during the current study are available in the Zenodo repository, [https://doi.org/10.5281/zenodo.5895163]”.

## Abbreviations

RR: Relative risk
RTC: Randomized controlled trial
OR: Odds ratio
CAP: Community‐acquired pneumonia
WTP: Willingness-to-pay
QALY: Quality adjusted life years
ICER: Incremental cost effectiveness ratio
